# Benefits and Challenges with Applying Unique Molecular Identifiers in Next Generation Sequencing to Detect Low Frequency Mutations

**DOI:** 10.1371/journal.pone.0146638

**Published:** 2016-01-11

**Authors:** Ruqin Kou, Ham Lam, Hairong Duan, Li Ye, Narisra Jongkam, Weizhi Chen, Shifang Zhang, Shihong Li

**Affiliations:** 1 Department of Development, GENEWIZ LLC, 115 Corporate Blvd., South Plainfield, NJ, 07080, United States of America; 2 Department of Bioinformatics, GENEWIZ CN, 218 Xinghu Street, Suzhou, Jiangsu, 215123, China; The University of Hong Kong, HONG KONG

## Abstract

Indexing individual template molecules with a unique identifier (UID) before PCR and deep sequencing is promising for detecting low frequency mutations, as true mutations could be distinguished from PCR errors or sequencing errors based on consensus among reads sharing same index. In an effort to develop a robust assay to detect from urine low-abundant bladder cancer cells carrying well-documented mutations, we have tested the idea first on a set of mock templates, with wild type and known mutants mixed at defined ratios. We have measured the combined error rate for PCR and Illumina sequencing at each nucleotide position of three exons, and demonstrated the power of a UID in distinguishing and correcting errors. In addition, we have demonstrated that PCR sampling bias, rather than PCR errors, challenges the UID-deep sequencing method in faithfully detecting low frequency mutation.

## Introduction

Early diagnosis is often the key in disease management. For a tumor made up of a heterogeneous cell population each with its own set of somatic mutations, the ability to detect a small population of tumor cells with characteristic driver mutations is important to predict prognosis and tailor with effective therapy [1–2]. For body fluid carrying a few exfoliated or circulating tumor cells among a majority of normal cells, the ability to detect mutations specific to the tumor cells holds promise for non-invasive early diagnosis of new cases and painless follow-up of residual diseases [3]. For infectious diseases with a complex population of viral pathogens, the ability to detect low-abundant drug-resistant variants can significantly impact the treatment outcome[4–5]. Advancements in next generation sequencing (NGS) has made it possible to detect low occurrence mutations in a heterogeneous population [6]. The potential of NGS deep sequencing, however, was hampered by systemic errors of PCR and sequencing methods [7–9]. Molecular indexing combined with deep sequencing holds great promise to break the limit imposed by PCR and sequencing errors, and enables the detection of rare and ultra-rare mutations [10–13].

Tagging individual templates with a molecular barcode has been proposed and reported since 2007 [10–16]. The molecular barcodes or molecular indexes have been given various names, such as unique identifiers (UID), unique molecular identifiers (UMI), primer ID, duplex barcodes, etc. They are usually designed as a string of totally random nucleotides (such as NNNNNNN), partially degenerate nucleotides (such as NNNRNYN), or defined nucleotides (when template molecules are limited). UID or UMI are introduced to targeted templates by ligation or through primers during PCR or reverse transcription. Tagging DNA fragments with UIDs or duplex barcodes has been shown to reduce errors and improve sequencing accuracy [10, 17]. Tagging viral RNA with primer ID or immunoglobulin mRNA with UMI has been reported to overcome oversampling [12,18]. Tagging total transcriptome of a single cell has been shown to enable quantitative access of expression level of individual genes in individual cells [19].

For low frequency mutation detection on the defined locus of a human genome, direct amplification of targeted locus with a UID-incorporated primer, as described by Kinde et al, is most straightforward. We tested the approach on a set of mock templates whose sequences were validated by the Sanger method. We have confirmed the power of UIDs in distinguishing true mutation from error occurring during PCR amplification and Illumina sequencing, and measured the combined error rate for PCR and Illumina sequencing at each nucleotide position of an exon. The average combined error rate of 25 cycles of PCR and Illumina sequencing, ranges from1.2–2.5 per 1000 bps depending on the DNA Polymerases used in PCR, and is correctable by UID consensus. Rather than PCR error, PCR sampling efficiency and sampling bias challenge the application of the method in detecting rare mutations faithfully at its true frequency.

## Materials and Methods

### 1. Mock DNA

7 exons each with known mutations were selected based on their frequent appearance in bladder cancer. Wild type and mutant fragments of *PIK3CA* Exons 9 and 20, *HRAS* Exons 3 and 7, as well as *FGFR3* Exons 7, 9 and 14 were synthesized individually, cloned into pUC57, and confirmed by Sanger sequencing. The mutants included R248C in Exon 7, Y373C in Exon 9, K650T and K653H in Exon 14 of *FGFR3*, G13V in Exon 1, K117N in Exon 3 of *HRAS*, E542K in Exon 9, and H1047L in Exon 20 of *PIK3CA* (ref to Table 1). In addition, we retained a few other gene synthesis errors as examples of deletion type mutations. The confirmed plasmids were transformed back to *E*. *coli*, and bulk DNA was prepared from individual colonies and re- confirmed by Sanger sequencing.

**Table 1:** List of mock genes.

Mock Gene Description	SNP and Chromosome position
FGFR3_E7_WT (112 bp)	Chr4:1803563–1803674
FGFR3_E7_R248C	Chr4:1803564 G>A
FGFR3_E9_WT (129bp)	Chr4:1806085–1806214
FGFR3_E9_Y373C	Chr4:1806099 T >C
FGFR3_E14_”WT” (114 bp)	Chr4:1807819–1807932, 1807895A>C
FGFR3_E14_N653H	Chr4:1807898A>C and 1807895A>C
FGFR3_E14_K650T	Chr4:1807889A>C
PIK3CA_E9_WT (86 bp)	Chr3:178936024–178936110
PIK3CA_E9_E542K	Chr3:178936083 C>T
PIK3CA_E20_WT (118 bp)	Chr3:178952017–178952135
PIK3CA_E20_H1047L	Chr3:178952085A>G
HRAS_E1_WT (120bp)	Chr11:534221–534351
HRAS_E1_G13V	Chr11:534285G>T
HRAS_E3_WT (119 bp)	Chr11:533480–533599
HRAS_E3_K117N	Chr11:533552C>G

### 2. Primer design

Gene-specific PCR primers were designed on Ion AmpliSeq^™^ Designer (Life Tech, USA), using the parameter for multiplexing. The GSP primers were extended with a stretch of random nucleotides as UIDs, and partial P5 or P7 adaptors to facilitate barcoded libraries construction for Illumina sequencing. In some cases, 22 nucleotide UID was embedded in the forward primer only; in other cases, 6–12 nucleotide UID was embedded in both forward and reverse primers. The primer pairs were tested individually before multiplexing.

### 3. UID assignment

Each strand of a double stranded template was assigned with one UID during the 2 cycles of the 1^st^ stage of PCR with gene-specific primers extended with UID and adaptor. The PCR was initially performed in a 20 μL system containing 10 μL of Platinum^®^ 2x Multiplex PCR Master Mix, 2 μL of 2 μM primer pool and 0.01 to 1 ng each templates. The mixture was denatured for 5 min at 95°C, amplified in 2 cycles of 3-step PCR (30s at 95°C, 90s at 60°C and 30s at 72°C) and further incubated for 5 min at 72°C. In later experiments, 10 μL of NEBNext^®^ High-Fidelity 2 x PCR Master Mix (with Q5 high fidelity enzyme) was used, and the reaction was supplemented with 2 μL of 10x Taq buffer, 0.6 μL of 50 mM MgCl_2_, 0.04 μL of Platinum Taq polymerase, and carried out at modified conditions (5 min at 98°C, 2 cycles of 30s at 98°C, 90s at 60°C and 30s at 72°C and final 5 min at 72°C).

### 4. UID primer removal

The above Stage 1 PCR reaction mix was digested with 2 μl exonuclease I (20 unit/μl, NEB) at 37°C for 60 minutes and the products were purified using magnetic beads (Agencourt AMPureXP, Beckman Coulter, Inc). The purified products were used as templates for the 2^nd^ stage PCR.

### 5. Library construction

Stage 2 PCR was carried out in a 50 μL system containing 25 μL of Platinum^®^ 2x Multiplex PCR Master Mix, and 5 μL of Illumina barcoded primers. The PCR program included 5 min at 95°C, 20–30 cycles of 3-step amplification (30s at 95°C, 90s at 60°C and 30s at 72°C) and final 5 min at 72°C. Similarly, 25 μL of NEBNext^®^ High-Fidelity 2X PCR Master Mix (with Q5 enzyme) was used in later experiments for higher fidelity, supplemented with 5 μL of 10x Taq buffer, 1.5 μL of 50 mM MgCl_2_ and 0.1 μL of Platinum Taq polymerase to maintain uniformity. PCR conditions were modified to 5 min at 98°C, 30 cycles of 30s at 98°C, 90s at 60°C and 30s at 72°C and final 5 min at 72°C. The products were purified using magnetic beads and quantified using Qubit 2.0 Fluorometer (Life Technologies, Carlsbad, California, US).

### 6. Library QC and quantitation

Library quality, quantity and size were further examined with Agilent 2100 Bioanalyzer (Agilent Life Science, Santa Clara, California, US). In general, 20–100 ng DNA library with size around 340 bp were obtained. After quality controls, barcoded libraries from different samples were pooled and sequenced in both forward and reverse directions on Illumina MiSeq platform using 2x 150 bp chemistry.

### 7. Data analysis

Miseq reads Fastq data were QC filtered according to standard Illumina criteria. Sequencing primers and adaptors were trimmed along with terminal nucleotides with Q scores below 30. The quality filtered paired reads were assembled using Pandaseq, and the assembled reads were grouped based on the UID, and mapped to reference genes. UID was identified as nucleotides between the adaptor and targeted amplicons. UIDs that differed by up to 2 out of 22 nucleotides, or differed by 1 out of 12 nucleotides, were clustered and regarded as one.

### 8. Statistics

To estimate if two closely related UIDs are two independent UIDs or derived from one another via PCR error, we used the Binomial distribution model and Poisson distribution model respectively. The probability that two randomly sampled UIDs were independent with n nucleotides and differ at k positions is given by P(k) = Binom(k,n,0.75). When UID contains 22 nucleotides, the chance of any two UIDs differ by 1 nucleotide is given by, P(1) = Binom(1, 22, 0.75) = 3.75E-12; the chance of any two UIDs differ by 2 nucleotides is given by P(2) = Binom(2, 22, 0.75) = 1.18E-10. The probability that one UID is mutated from the other by PCR error is given by Poisson (λ, x). Assuming PCR error rate is 0.001, the expected mutation in 22 bps will be 22*0.001 = 0.022. The probability of having a related UID with 1 base mutation is P(0.022, 1) = 0.0215; the probability of having a related UID with 2 base mutation is P(0.022, 2) = 2.37E-4. The probability of two related UIDs derived from each other via PCR error far exceeds the probability of being independent. Therefore UIDs are clustered first and those differed by up to 2 nucleotides were counted as one.

## Results

### 1. Experimental design and working principle

**Fig 1:**
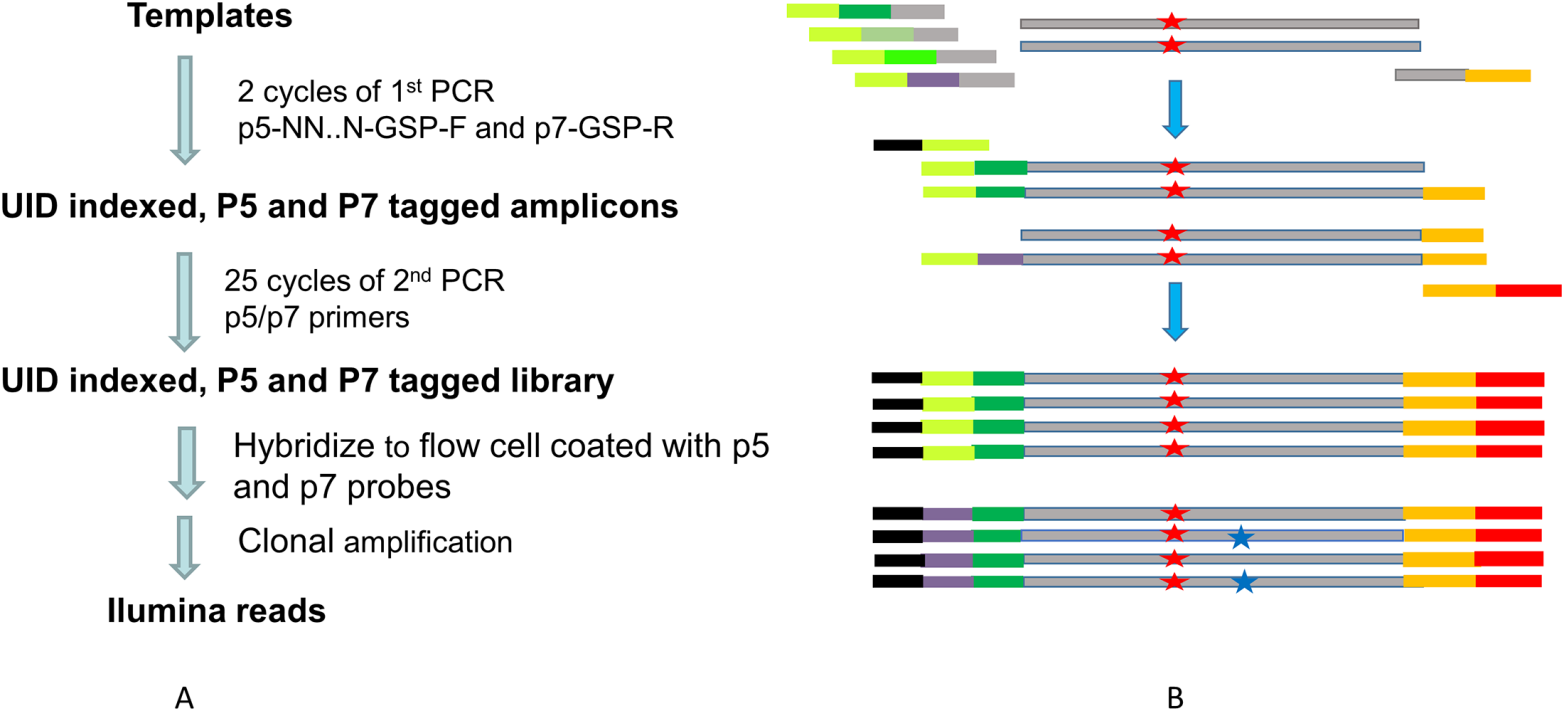
UID-targeted DNA sequencing workflow and the principle in distinguishing errors from true mutation. (A) Illustration of UID-targeted DNA sequencing workflow. (B) True mutation from errors introduced during PCR and sequencing. A true mutation (illustrated as red star) is expected to be present in all the reads carrying the same UID (or derived from the same template molecule), while an error (illustrated as blue star) is expected in some but not all the reads carrying the same UID.

**Fig 2:**
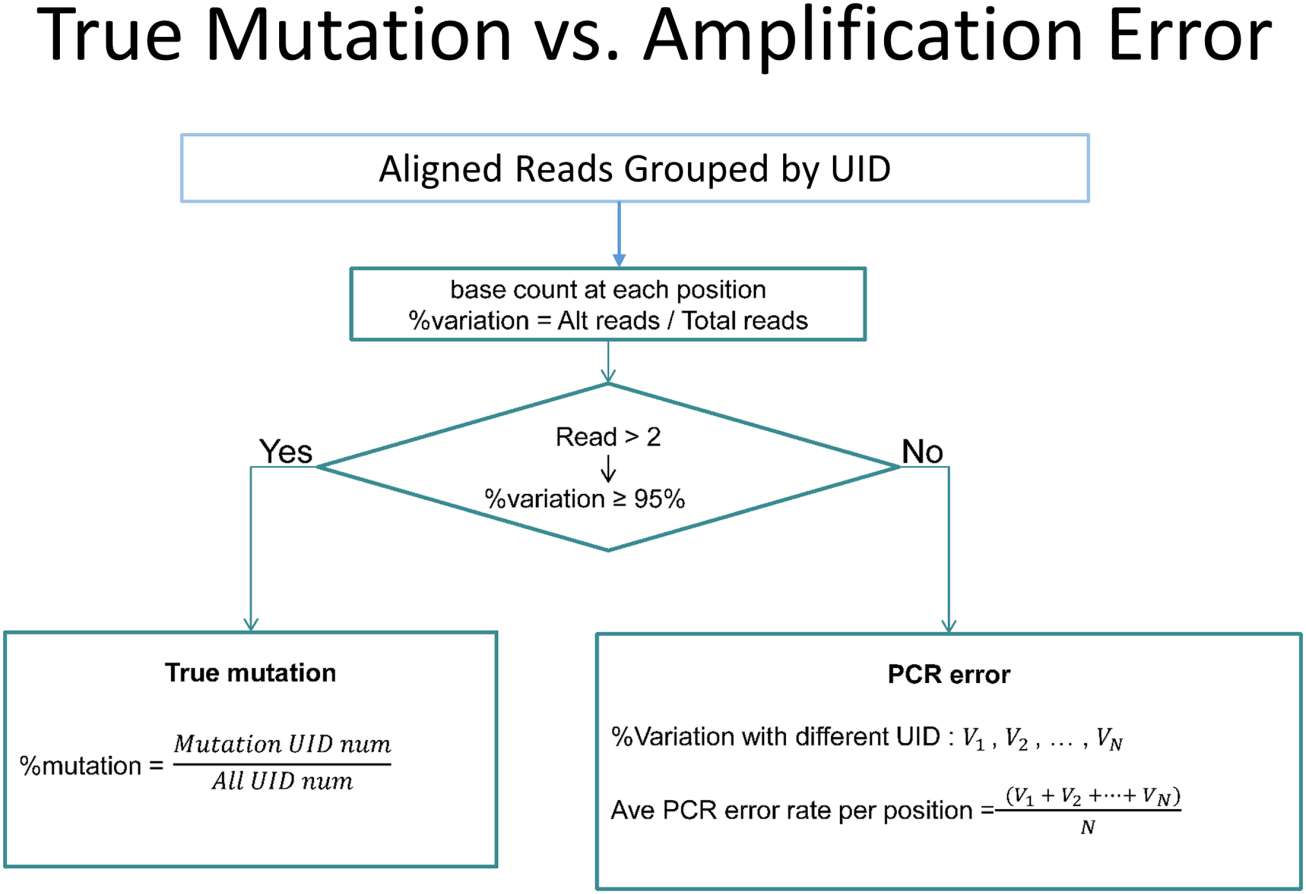
Flowchart for error vs mutation data analysis. Reads were grouped by UID. When an UID has 3 or more reads, the ratio of altered reads/total reads was calculated. If the ratio was more than 95%, the altered nucleotides were counted as pre-existed in the template tagged with the UID; if the ratio was less than 95%, the altered nucleotides were counted as error occurred during the amplification of the tagged template or the sequencing step.

Templates were amplified in a two-stage PCR. The first stage included 2 cycles of PCR with gene-specific primer extended with UID and partial adaptor. During the 2 cycles of the initial PCR step, each strand of a double stranded molecule was barcoded with one unique identification sequence and extended with an adaptor. The excess primers were digested with exonuclease I and products were beads purified. The UID-barcoded templates were then amplified with adaptor primers for 20–30 cycles and sequenced using Illumina 2*150bp chemistry. Fig 1 illustrates the work flow and experiment principles and Fig 2 shows the flowchart for error vs mutation data analysis.

For proof of concept, we employed mock templates. The mock templates included 7 exons, both wild type and mutants, chemically synthesized and cloned into pUC57. All plasmids were prepared from single colonies and validated by Sanger sequencing (Table 1). PCR primers were designed using parameters for multiplexing. The primer pairs were tested individually before multiplexing. Platinum enzymes were used initially for robustness and uniformity in multiplexing PCR. High fidelity Q5 enzymes with spiked in Platinum Taq enzyme were used later for improved fidelity.

### 2. UID design and false UID correction

We choose a stretch of random nucleotides as UID over a stretch of defined sequence for greater diversity the former offered. The number of N needed should be proportional to the numbers of template molecules. We tested short UID (6 to 12 random Ns) on each end of the amplicon or longer UID on one side of the amplicon only, and settled with 22 random Ns in the forward primer only. Our rationale is that the possible combination of 22 random nucleotides (4^22 = 1.76E+13) far exceeds the primer molecule number available in a 20 μl reaction with 200 nM primers (6.02E+23 * 20E-6 *200E-9 = 2.4E+12). Therefore, 22N is likely to ensure each template molecule obtains a unique combination of nucleotides regardless of the template number.

Given error occurs in both the PCR and the sequencing step, the nucleotides within UID have equal chance to accumulate mutation as the nucleotides flanked by UID. Error within UID may result in false UID, and leads to overestimation of tagged template molecules, false identification of mutations, or skewing of mutant representation. Table 2 shows a clusters of UIDs for FGFR14-Exon14, detecting altered nucleotides from reference at position1. There are 1098 reads under UID #24, and 1–9 reads under 6 related UIDs, each differs from #24 by a single nucleotide. The chance the 6 related UIDs represent independent templates was statistically low, while the chance that they are derived from UID#24 by PCR or sequencing error is much higher (ref to material and method, statistics). If we count UID #2124, 2162, 2074 and 2061 as true UIDs representing 4 independent templates, we would inflate the SNP 5 fold. Therefore, we have incorporated a step to cluster UIDs that differ by 1 or 2 nucleotides, and combined them into one single UID group.

**Table 2:** A UID Cluster with false UIDs derived from mutation of UID#24.

UID Rank	UID Alignment	Total Read Number	Alt Read Number
2124	GACATGTCT**C**CGTAGGTAATGC	4	4
2162	GACATGTCTTCGT**G**GGTAATGC	3	3
2074	GACATGTC**C**TCGTAGGTAATGC	9	9
**24**	GACATGTCTTCGTAGGTAATGC	**1098**	**1096**
2161	GACATGTCTTCGTAGG**C**AATGC	3	3
2347	GACATGTCTTCGTAGG**A**AATGC	1	1
2348	GACATGTCTTCGTAGG**G**AATGC	1	1

### 3. PCR and sequencing error rate was measured by UID method

For proof of concept, 1 ng pUC57-*FGFR3*-exon14, as a defined mixture of four different variants, was amplified in the 2-stage PCR and deep sequenced. After 2-stage PCR (using Platinum multiplex PCR master mix) and Illumina sequencing, the QC filtered reads were grouped according to UID. UIDs with 1 or 2 reads were left out. Any UIDs with 3 or more reads were clustered to remove false UIDs. In total, 2.4 million reads, grouped under 4190 authentic UIDs were used for analysis (S1 Table).

As false UIDs were removed after UID clustering, we are confident that all reads with the same UID came from the same template molecule. Under each UID, for each position of entire exon of 114 base pairs, we counted the number of reads matching the reference (ref), and the number of reads differing from the reference (alt), and calculated the ratio of alt/total. Table 3 is a snapshot of position1 (Chromosome 4: 1807819). For this particular position, 4910 templates (represented by unique UID, each with at least 3 reads) were sampled, and about 2000 of them contained error (shown as altered read), and the rest contained no error (for full information, ref to S1 Table). The Ratio of altered reads/total reads ranged from 0 to1.

**Table 3:** Nucleotide identities at chromosome 4: 1807819 (Position 1) in *FGFR3* Exon14 under individual UIDs.

UID Cluster	Ref	Alt	Total Read	Alt Reads	% Alt
GCCTGCTGTCGGGTAGTATGGC	C	T	1282	0	0.00%
GGCGTCCAGAATGACTATTTAT	C	T	1249	2	0.16%
GGCAAAGGCGCAGATAGTATAT	C	T	1231	5	0.41%
GTTTTGTGGTGGTACCTATTCT	C	T	1220	3	0.25%
TTTAATGTGGGCAAGGCGTGAA	C	T	1183	2	0.17%
TTAGTTGTGCGTGCGCGCGGTT	C	T	1175	3	0.26%
TTGTGGGTCGATATCGGATGAT	C	T	1174	1	0.09%
TTGCTGAGCAGTACTCGTGCCT	C	A	1165	4	0.34%
GACATGTCTTCGTAGGTAATGC	C	T	1117	1115	99.82%

If the ratio of altered reads/total reads was less than 1 (we set the threshold at 95%, as majority of the rows in Table 3), we considered the alteration as an error that occurred during the 2^nd^ stage PCR or Illumina sequencing, and recorded the ratio as combined error rate of PCR and sequencing. For each nucleotide, we recorded its error rate for each UID (or each template), and used the average as a combined error rate for the 25 cycles of stage 2 PCR and Illumina sequencing at the particular position. For Position1, the average error rate for all templates was calculated as 0.0022 or 2.2 error/1000bp/25 cycles PCR and Illumina sequencing.

**Fig 3:**
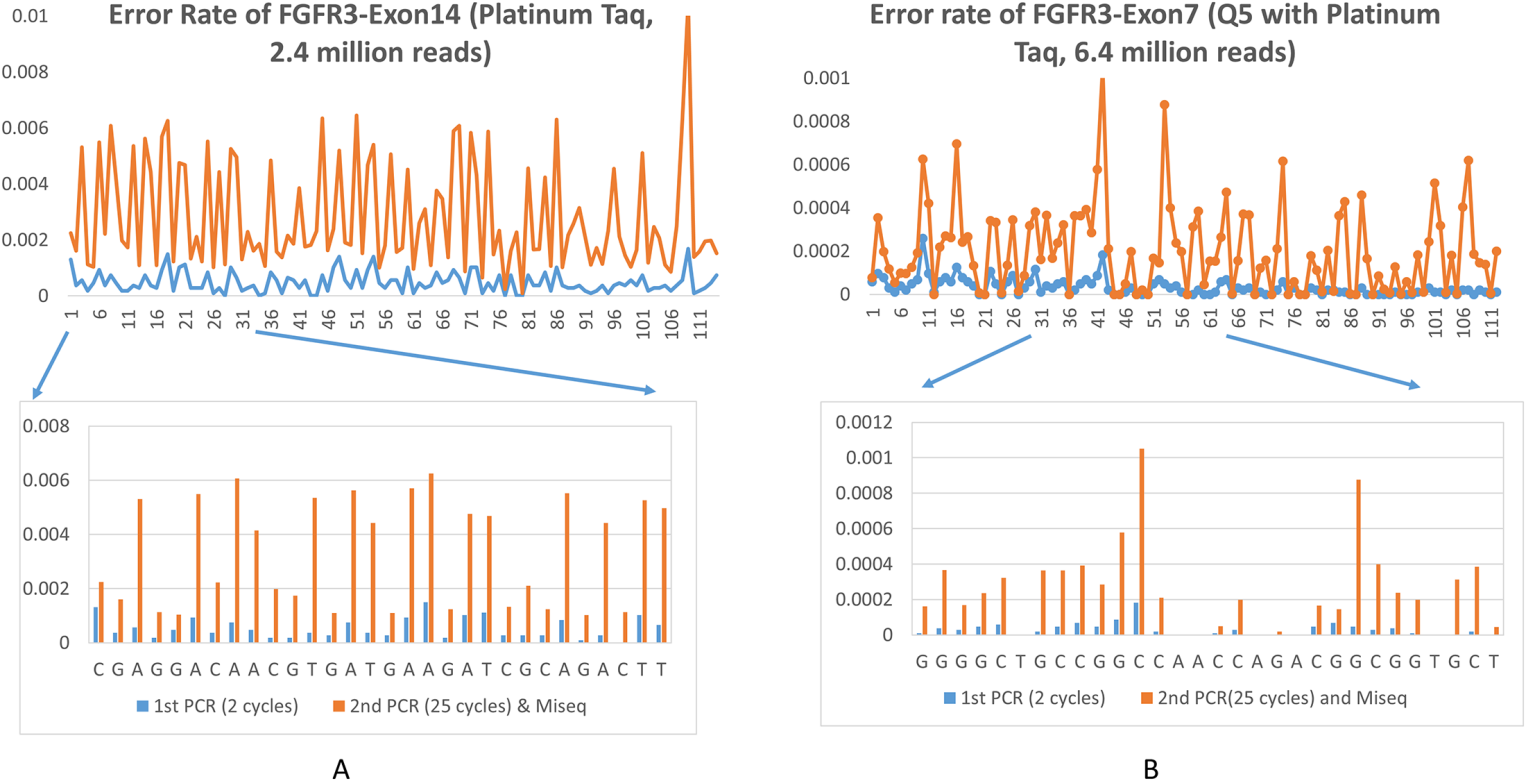
Error rate at each nucleotide position of *FGFR3*-Exon14 and *Exon 7*. (A) Error plotted for all 114 nucleotides of Exon14 (amplified with Platinum Taq), with 30 nucleotides magnified. (B) Error plotted for all 112 nucleotides of Exon7 (amplified with Q5 enzyme), with 30 nucleotides magnified.

If the ratio of alt/total equals 1 (we set the threshold at > or = 95%), we considered it real SNP or that the error occurred in Stage 1 during the 2 early PCR cycles (the 9^th^ and last row, selected from the rare events in Table 3). Since the template input at most positions was an all wild type prepared from a single colony and verified by Sanger sequencing, any alteration was counted as an error arose in the UID assigning Stage 1 PCR, rather than true mutation. For each nucleotide, we recorded the number of UIDs with all reads altered, and calculated the ratio of such UIDs (templates read wrong) to the total UID (total templates sampled), and counted this as PCR error for Stage 1 PCR. For Position 1, this error rate was averaged to 0.0013 or 1.3 errors per 1000 bps. Fig 3 shows the error rate of each of the 114 positons of *FGFR3*-exon 14, with the 1^st^ PCR error rate accounting for the initial 2 cycles of PCR, and the 2^nd^ PCR error rate accounting for the latter 25 cycles of PCR and the Illumina sequencing.

**Fig 4:**
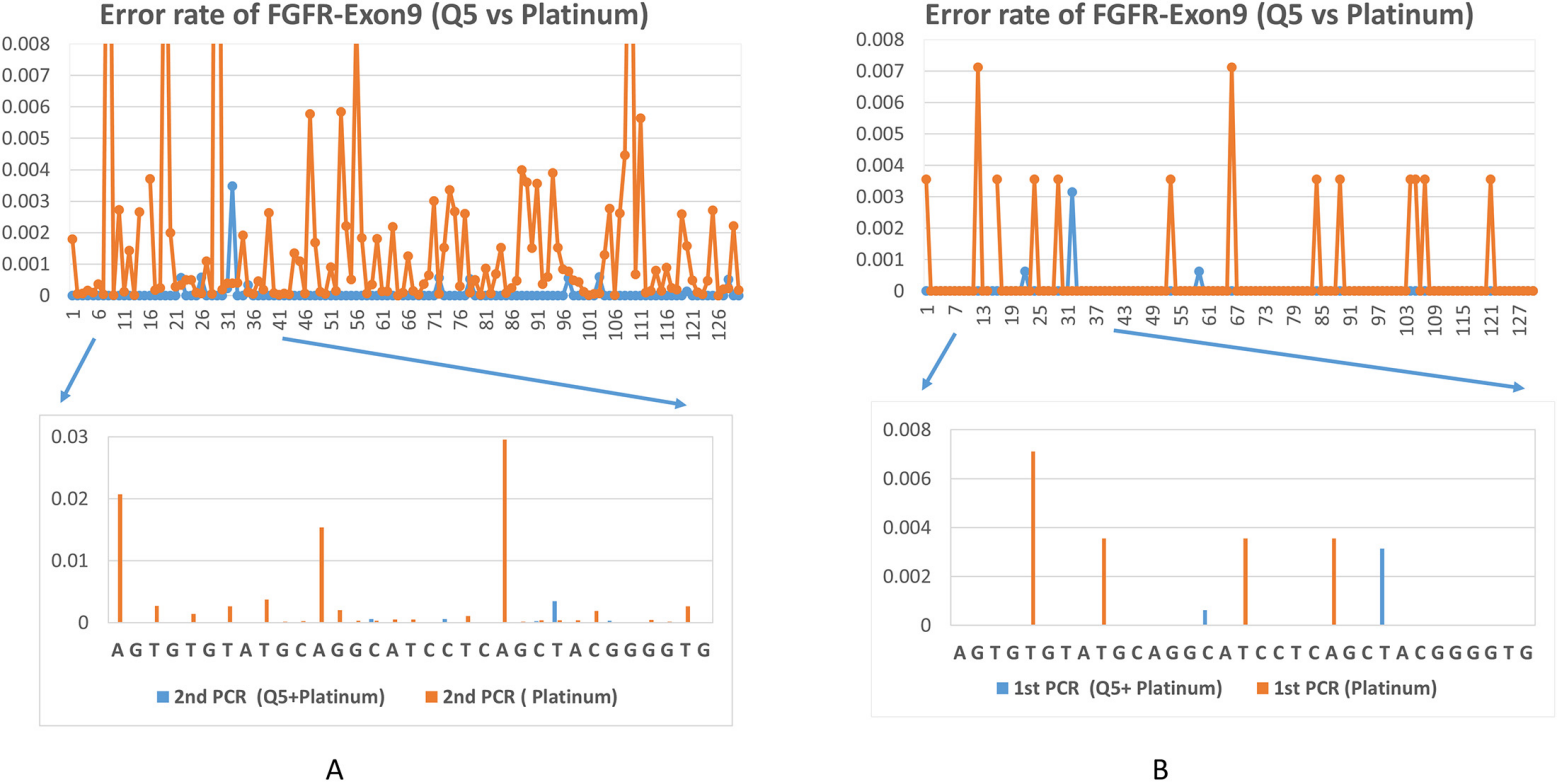
Q5 DNA polymerase improved the fidelity of UID deep sequencing. (A) Comparison of error rate at each position of FGFR3-Exon 9 in Stage 2 PCR and Illumina sequencing when Q5 (with Taq spike in) vs Platinum DNA Polymerase was used. (B) Comparison of error rate at each position of FGFR3-Exon 9 in Stage 1 PCR when Q5 (with Taq spike in) vs Platinum DNA Polymerase was used.

As shown in Table 4, we calculated the error rate of the 114 nucleotides of FGFR-Exon14 and the 130 nucleotides of Exon9. The average error rate of stage 2 PCR (25 cycles) and Illumina sequencing ranges from 0.0017 to 0.0028. The average error rate of Stage 1 PCR (2 cycles) is about 4 folder lower and ranges from 0.0004 to 0.0005. The number is well within the ranges of Taq polymerase’s fidelity, and consistent with others’ observations [10]. The errors occurred during stage 2 PCR and Illumina sequencing step are correctable by UID consensus. The errors occurred during stage 1 PCR, however, could not be distinguished from true mutations. Thus, we substituted the Platinum-based PCR mix with high fidelity Q5-based reaction mix (we spiked in Taq DNA polymerase as described in the material and methods to increase robustness). The error rate for the above two exons, and one additional exon, FGFR3-Exon7, were calculated. As expected, Q5 DNA polymerase further reduced the average error rate by a log or so (Table 4 and Figs 3–4). The error rate with Platinum Taq vs Q5 was plotted in the same figure for FGFR3-Exon9, as they were based on similar amount of reads.

**Table 4:** PCR error rate on 3 exons.

	Platinum Taq		Q5 with Platinum Taq		
	FGFR3-E14	FGFR3-E9	FGFR3-E14	FGFR3-E9	FGFR-E7
1st PCR Average	4.83E-04	4.14E-04	9.40E-05	3.39E-05	3.02E-05
2nd PCR Average	2.85E-03	1.68E-03	9.08E-05	6.22E-05	2.01E-04
1st PCR Stdev	3.61E-04	1.31E-03	5.34E-04	2.86E-04	3.97E-05
2nd PCR Stdev	1.92E-03	3.83E-03	4.80E-04	3.29E-04	1.99E-04
**Read#**	**2400000**	**90059**	**62400**	**54638**	**6440196**
UID#	4910	281	997	1589	104094

**Fig 5:**
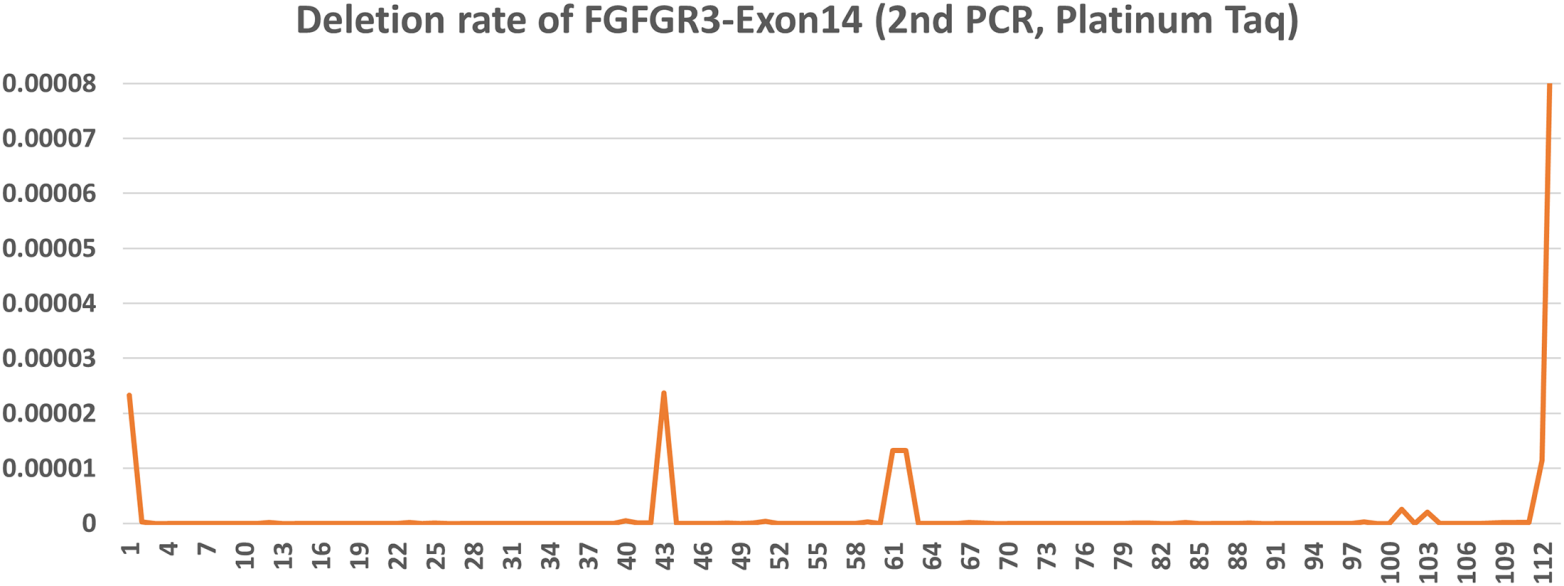
Error rates of deletion types in *FGFR3* Exon14. Platinum DNA polymerase was used. Notice the scale is 100 fold lower than that of Fig 3 or Fig 4.

The error rate varied among the three exons (Table 4) and at each position within each exon (Figs 3 and 4). As expected, the errors were more evenly distributed and the error rate was less fluctuated with higher read depth, such as in the case of FGFR3-E14 (Platinum, 2.4 million reads) and E7 (Q5, 6.4 million reads). With Platinum Taq enzyme (a type I polymerase), the errors were in general substitution type, and the error rate was generally higher at A or T than at G or C (Figs 3a and 4a). With the Q5 enzyme, a type II polymerase, the higher error rate did not correlate with A/T sites (Fig 3b). Insertion or deletion errors rarely happen, as show in Fig 5. Excluding the ends of the amplicon, the highest deletion rate is 0.00002, or 2 deletion events per 100,000 bp per 25 cycles. The relatively higher deletion rate at the two ends of the amplicon were artifact from UID trimming. For substitution errors, transitions from C to T, T to C, G to A, and A to G dominated over other types of errors. This was consistent with others’ observations on Taq polymerase [20–21]. For example, the errors occurred at Position 1 (Chromosome 4: 1807819) were predominantly C to T. C to A, or C to G happened but rarely.

### 4. PCR sampling efficiency and sampling bias challenged the reliability of the UID method in detecting low frequency mutations

In the above experiment for FGFR3-Exon14, 1 ng or about 3 million copies of pUC57- *FGFR3*-Exon14 were used as templates. Of the 1 ng DNA template or close to 3 million molecules, 97% contain A>C change at both position 1807895 and 1807898 of Chromosome 4, 1% contain A>C mutation at position 1807898 only (N653H), 1% contain A>C at1807889 (K650T), 1% contain ACG deletion at 1807863–1807865, and 0.01% contain G>T changes at 1807823. Table 5 compared the input (templates included and not included) and output (templates read or misread as represented by UID number). The double mutant mixed in 97% was detected at 99.6%, while the other three mutants mixed in at 1% of the population were detected at a lower percentage of 0.02% (Chr4:1807863–1807865 del:ACG), or totally missed (Chr4: 1807889 A>C, or Chr4:1807895 A>C). Additional variants, although not among the input, were also detected, and some at up to 0.26%, representing errors occurring in Stage 1 PCR.

**Table 5:** Comparison of template input with the sequencing output for pUC57-*FGFR3*-E14.

Templates	Input (% total)	READ#	Output (based on reads)	UID #	Output (based on UID)
Chr4: 1807895 A>C and 1807898 A>C	96.9%	2406654	99.60%	4174	99.59%
Chr4:1807895 A>C	1%	597	0.02%	0	0%
Chr4: 1807889 A>C (K650T)	1%	150	0.01%	0	0%
Chr4: 1807889 A>G or T, 1807895 A>C and 1807898	0%	5796	0.24%	11	0.26%
Chr4:1807863–1807865 del:ACG, & 1807895 A>C	1%	30	0.00%	1	0.02%
Chr4: 1807823 G>T, 1807895 A>C and 1807898 A>C	0.01%	554	0.02%	1	0.02%
Chr4: 1807823 G>A, 1807895 A>C and 1807898 A>C	0%	2480	0.10%	4	0.10%

1 ng input, or 2783–2694606 copies of plasmid variants, were used as templates. Platinum DNA polymerase was used for both Stage 1 and Stage 2 amplification.

It appeared that for templates that made up the majority of the population, the sequence output was close to the template input. However, for templates that made up 1% or less of the population, the readout was far from accurate. Similar observations were made on the other exons (Table 6). HRAS-E1G13V, mixed in at 1%, was detected at 2.3%. The other variants mixed in at 0.1–1% were either missed or detected at a much higher percentage. UID correction effect was significant for two exons. Wild type FGFRR3-E9, mixed in at 1%, was detected at 3.6%. Without UID correction, it would be inflated to 21.5%. Similarly, mutant PIK3CA-E20, mixed in at 0.1%, was detected at 3.4%. Without UID correction, it would be 18.3%.

**Table 6:** Comparison of template input with the sequencing output for 5 more exons.

Template	Input	Read#	Output (based on reads)	UID #	Output (based on UID)
FGFR3-E7 WT	99.9%	6669968	100%	177998	100%
FGFR3-E7 R248C(Chr4:1803564 G>A)	0.1%	1	0%	0	0%
FGFR3-E9 Y373C(chr4:1806099 T >C)	99.0%	70722	69.0%	271	96.4%
FGFR3-E9 WT	1.0%	19336	21.5%	10	3.6%
HRAS-E1 WT	99.0%	265986	97.9%	1259	97.7%
HRAS-E1 G13V(Chr11:534285G>T)	1.0%	5753	2.1%	29	2.3%
PIK3CA-E20 WT	99.9%	45767	81.7%	768	96.6%
PIK3CA-E20 H1047L(Chr3:178952085A>G)	0.1%	10231	18.3%	27	3.4%
PIK3CA-E9 WT	99.9%	13390	100%	211	100%
PIK3CA-E9 E542K(Chr3:178936083 C>T)	0.1%	65	0%	0	0%

The experiment was repeated. In the subsequent experiment described below, high fidelity Q5 multiplex PCR master mix (with Platinum Taq enzyme spike in) was used instead of lower fidelity Platinum multiplex system. 7 pairs of plasmids, 0.1 ng or 270,000 molecules for each pair, were used as a template. Each pair was made up of 99.9% wild type molecules and 0.1% mutant with single nucleotide change. Again we tabulated UID numbers for wild types and mutants, and compared the output with the input. As shown in Table 7, the output is not accurate either.

**Table 7:** Sequencing output for 7 pairs of templates, each with 0.1% mutant input.

Template	Input	Read #	Output (based on reads)	UID #	Output (based on UID)
FGFR3-E7 WT	99.9%	6569826	99.998%	104094	99.99%
FGFR3-E7 R248C (Chr4:1803564 G>A)	0.1%	122	0.002%	9	0.009%
FGFR3-E9 WT	99.9%	54561	100%	1589	100%
FGFR3-E7 Y373C (chr4:1806099 T >C)	0.1%	0	0%	0	0%
FGFR3-E14 WT	99.9%	70256	100%	997	100%
FGFR3-E14 N653H (Chr4: 1807898 A>C)	0.1%	185	0%	0	0%
HRAS-E1 WT	99.9%	133233	100%	4856	100%
HRAS-E1 G13V (Chr11:534285G>T)	0.1%	0	0%	0	0%
HRAS-E3 WT	99.0%	111253	100%	2476	100%
HRAS-E3 K117N (Chr11:533552C>G)	0.1%	0	0%	0	0%
PIK3CA-E20 WT	99.9%	13878	99%	594	99%
PIK3CA-E20 H1047L (Chr3:178952085A>G)	0.1%	110	0.8%	6	1.0%
PIK3CA-E9 WT	99.9%	57895	100%	2494	100%
PIK3CA-E9 E542K (Chr3:178936083 C>T)	0.1%	0	0%	0	0%

0.1 ng input, or 291850 plasmid molecules with 291 mutant copies, were used as templates. High fidelity Q5 enzymes with spike-in Taq DNA polymerase was used for both Stage 1 and Stage 2 amplification.

Of the 7 template genes each starting with 0.1% mutant, mutants were not detected in 5 genes, under-represented in 1 gene, and over-represented in 1 gene. The over-representation, 10 fold for *PIK3CA*-E20, could be due to under-sampling. Reads on PIK3CA all belong to 594 UID groups, indicating they are from 594 template molecules. In other words, only 0.2% of the 291,850 template molecules were effectively sequenced (Table 8). Similarly, the failure to detect the 0.1% mutant in *FGFR3*-E9, E14, *HRAS*-E1, E3, and *PIK3CA*-E9 could also be due to under-sampling as only 997–4856 templates, or 0.3% -1.7% of the 291,850 template molecules were sequenced. However, the 11 fold under representation of the mutant in *FGFR3*-E7 could not be due to under-sampling alone as we have obtained sufficient reads from a sufficient number of template molecules.

**Table 8:** PCR sampling efficiency for each genes in multiplex PCR.

	Template #	Reads #	UID#	% Template Sampled
**FGFR3-E14**	291850	64472	997	0.30%
**FGFR3-E7**	291850	6569826	104094	35.60%
**FGFR3-E9**	291850	57895	1589	0.50%
**HRAS-E1**	291850	133233	4856	1.70%
**HRAS-E3**	291850	111253	2476	0.80%
**PIK3CA-E20**	291850	13878	594	0.20%
**PIK3CA-E9**	291850	57895	2494	0.90%

0.1 ng input or 291850 copies of plasmid molecules was used as templates. High fidelity Q5 enzymes with spike-in Taq DNA polymerase was used for both Stage 1 and Stage 2 amplification.

**Fig 6:**
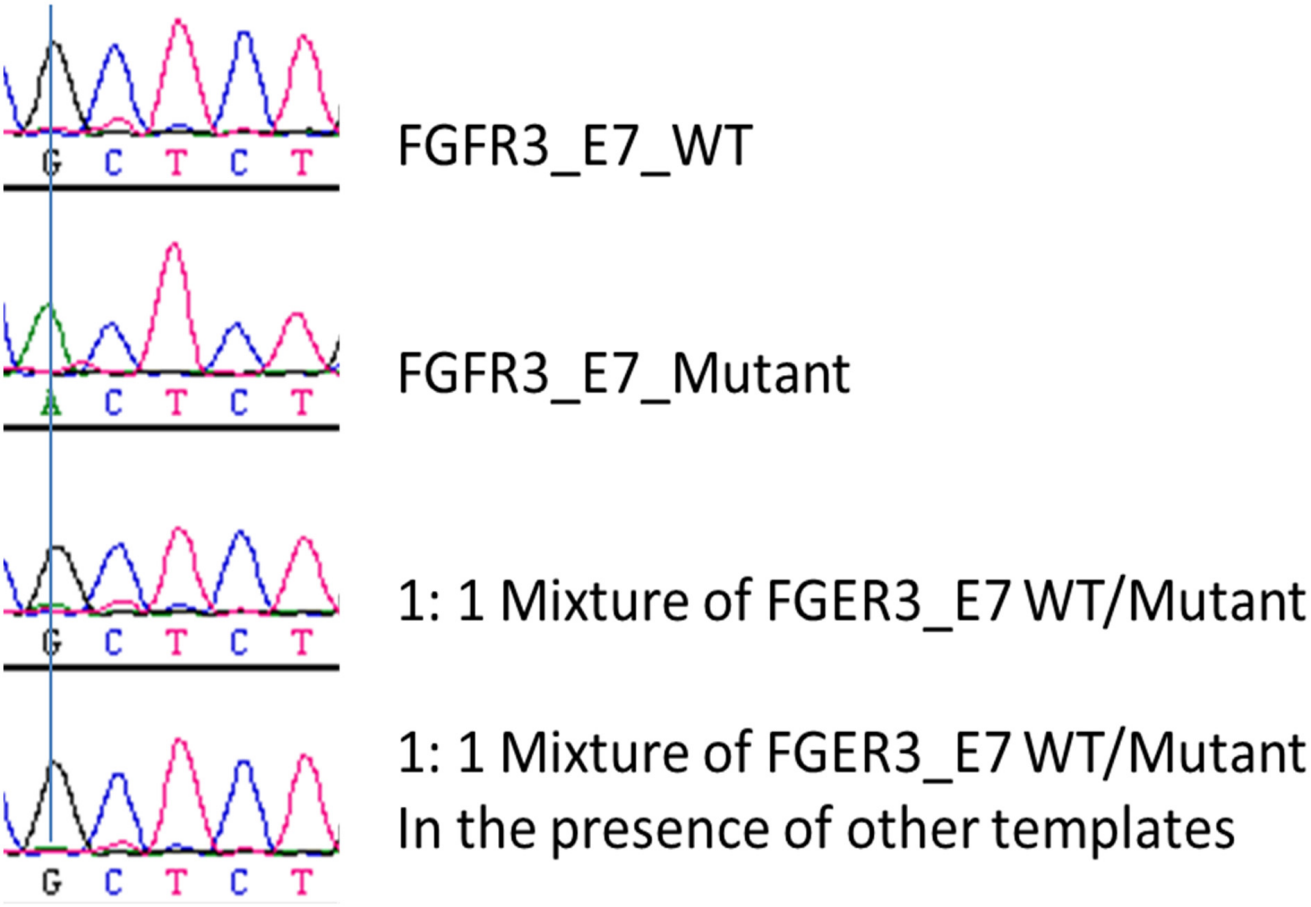
PCR Bias for wild type over mutant FGFR3-Exon7. FGFR3-Exon7 was amplified from the wild type template, mutant template (Chr4:1803564 G>A), or 1:1 mixture of the wild type and mutant templates, and the PCR products were sequenced by Sanger method.

FGFR3-Exon7 has been sequenced in sufficient depth with over 6 million reads generated in two repeated experiments. In both experiments, the PCR amplification was greatly biased for wild type templates. It is hard to understand, but it appears that mutants were suppressed, while wild type templates were preferentially amplified. To confirm the observation, we PCR amplified FGFR3-exon7 from wild type template, mutant template, a mixture of WT and Mutant template at 1:1 ratio, and a mixture of WT and mutant templates in presence of the other 6 exons respectively, and Sanger-sequenced the PCR products. Fig 6 shows a sequence chromatogram of the correlating position. We were expecting a single “G” peak from the wild type template, a single “A” peak from the mutant template, and double peaks of “A” and “G” from the 1:1 mixture. To our amazement, we observed wild type sequence only from the 1:1 mixtures of wild type and mutant. The mutant that constitutes 50% of the template was not detected by Sanger method. So it’s not surprising that the same mutant constituting of 0.1% of the population was under-detected by deep sequencing.

The uncorrectable error rate with Q5 enzyme in this run was very low, with an average of 0, 39 errors per 100,000 bps for FGFR3-Exon7, 9, and 14 (Table 4 and Fig 4). Therefore, we concluded that PCR sampling efficiency and PCR sampling bias, more than PCR error, challenged the accurate detection of low frequency mutation in a heterogeneous population. PCR sampling efficiency and PCR bias among different genes could be resolved by performing individual PCR instead of multiplex PCR. However, PCR bias between wild type and mutant templates of same genes could not be easily resolved.

## Discussion

High fidelity DNA polymerase and the power of UIDs in distinguishing errors from true mutations make it possible in theory to detect mutation at 1% or lower frequency. In reality, however, under-sampling and sampling bias challenges the practical application of UID-deep sequencing. Only two cycles of PCR were performed to guarantee individual templates obtain no more than one unique UID per strand. Under-sampling would result when PCR efficiency and uniformity were not optimal to generate a sufficient and representative UID-tagged amplicons pool for later amplification. Primer-dimer, a common issue for PCR, was more prominent when longer UIDs were used or when UIDs were embedded in both forward and reverse primers. Removal of primer-dimer was often accompanied with loss of tagged products, and further contributing to under-sampling.

Both Stage 1 and Stage 2 PCR could be highly biased, as evidenced by sampling efficiency of different mock genes, under- or over-representation of mutant population within the same genes, and the wide ranges of read number per UID. A long stretch of random nucleotides, or UID itself, may also contribute to PCR bias directly. Some combination of the random nucleotides could hybridize to targeted templates, block their amplification, and contribute to the gene to gene bias; some combination of the random nucleotides could also hybridize to mutant or wild type genes at different efficiency and contribute to WT to mutant bias within the same genes.

The power of UID in distinguishing error from mutation promises to revolutionize early diagnosis of many diseases in a non-invasive fashion. However, to realize its full potential and turn the idea into robust and cost-effective diagnosis assays, many challenges need to be overcome. For a given template molecule, UID enables us to distinguish true mutation from errors. However, to accurately assess the mutant representation in a pool of templates, all the templates within the pool have to be uniformly tagged, no error shall occur during the tagging process, and the tags have to be immune to mutation in subsequent amplification. As we have discovered, 2 cycles of PCR was neither efficient nor uniform in tagging the template population, and the UID tags were prone to PCR error later in amplification just as the templates they flanked.

Similarly, tagging templates during reverse transcription and 1^st^ strand synthesis were equally challenged. As reported by Brodin et al [22], uneven frequency of resampling was one major issue in their attempt to improve sequencing accuracy of HIV viral population using primer IDs. Tagging template by ligation could be error free but ligation efficiency and uniformity has yet to be demonstrated.

For mutation detection in a large genome such as the human genome, the limit is also set by the availability of mutant templates. For a typical PCR reaction, 10–50 ng genomic DNA is used. About 33 ng human genomic DNA translates to 10,000 copies of double strand DNA. For mutation in a single copy gene, 1% means a meager 100 copies. A technique breakthrough is needed to efficiently and uniformly tag the entire template population to realize the full potential of molecular indexing. As of current, Digital PCR might be a better choice.

## Supporting Information

S1 Table UID and reads for FGFR3-E14. All UID clusters were listed by consensus sequence, total read number, and alteration rate at each nucleotide position of the amplicon.
(XLSX)

S2 Table UID and reads for FGFR3-E9. All UID clusters were listed by consensus sequence, total read number, and alteration rate at each nucleotide position of the amplicon.
(XLSX)

S3 Table UID and reads for FGFR3-E7. Only UID clusters with altered reads (3406 in total) were listed. Error-free UID clusters (100,688 in total) and singleton UID clusters (with 1–2 reads per UID cluster, 193,064) were not listed.
(XLSX)
